# 80 Establish a Prognostic Prediction Model in High-Risk Burn Patients by Artificial Intelligence

**DOI:** 10.1093/jbcr/iraf019.080

**Published:** 2025-04-01

**Authors:** Chun Chia Chen

**Affiliations:** Chung Shan Medical University Hospital

## Abstract

**Introduction:**

Burn injuries, particularly in high-risk patients, often lead to complex medical management, including graft surgery and extended hospital stays. Traditional tools like the Baux score are commonly used to predict mortality, with sensitivity ranging from 12% to 96% and specificity from 80% to 100%, depending on the patient population. However, the Baux score provides limited insight into complications such as the need for graft surgery or prolonged hospitalization. This study aims to develop an artificial intelligence (AI) model to improve prognostic predictions in high-risk burn patients, evaluating outcomes like graft surgery, hospital stays, and adverse complications, and compare it to the effectiveness of the Baux score.

**Methods:**

A retrospective analysis of 224 burn patients admitted to Burn Center in Chi Mei Medical Hospital between 2010 and 2019 was conducted. The AI model incorporated 14 clinical features, including demographic data, burn severity, comorbidities, and laboratory results. Machine learning algorithms, such as random forest, LightGBM, and XGBoost, were used to predict three outcomes: the need for graft surgery, prolonged hospital stays (>14 days), and adverse complications. Model performance was evaluated using accuracy, sensitivity, specificity, and the area under the receiver operating characteristic curve (AUC).

**Results:**

The AI model showed superior performance compared to the Baux score in predicting graft surgery, hospital stays, and adverse complications. The random forest model demonstrated the highest AUC for predicting prolonged hospital stays (81.1%), far exceeding the Baux score’s moderate predictive capability (AUC ≈ 0.65) in past literatures review. Similarly, for graft surgery, the AI model achieved an AUC of 78.8%, outperforming the Baux score, which primarily focuses on mortality prediction. For adverse complications, the AI model reached an AUC of 87.2%, while the Baux score showed lower sensitivity and specificity in predicting non-mortality-related complications.

**Conclusions:**

The AI model provides a more comprehensive and accurate tool for predicting critical outcomes in high-risk burn patients, such as the need for graft surgery and prolonged hospital stays, compared to the traditional Baux score. Integrating AI models into clinical practice could significantly improve decision-making, optimize resource allocation, and enhance patient care. Future research should focus on validating this model across different populations to further refine its accuracy.

**Applicability of Research to Practice:**

Integrating the model into hospital systems allows real-time, data-driven decisions that enhance patient outcomes. Its scalability means it can be applied across different healthcare settings, standardizing burn care practices and improving the overall management of burn patients. This research aligns with precision medicine, promoting individualized and effective interventions.

**Funding for the Study:**

N/A

